# S11-5 Recruitment of physically inactive adults to participate in the standardized program Jackpot.fit. Which strategies within the health system work?

**DOI:** 10.1093/eurpub/ckad133.057

**Published:** 2023-09-11

**Authors:** Sylvia Titze, Bernhard Novak, Markus Schweiger

**Affiliations:** University of Graz, Institute of Human Movement Science, Sport and Health, Graz, Austria; University of Graz, Institute of Human Movement Science, Sport and Health, Graz, Austria; Social Security Service for Entrepreneurs, Graz, Austria

## Abstract

**Purpose:**

The Jackpot.fit program was developed by the Austrian health insurance funds and the organized sport with the aim of offering a standardized training for physically inactive adults. The first semester is free of charge. The original idea was that health insurance companies would use different strategies to recruit participants and that organized sports would offer the standardized program in local sports clubs. In this context, we wanted to investigate which recruitment strategies work well.

**Methods:**

In one region of the province of Styria (Austria), we wanted to offer a dense network of Jackpot.fit classes, as the sports program was to take place close to home. Adults who signed up for Jackpot.fit received an online questionnaire. One question asked: “How did you find out about Jackpot.fit?” Ten answers were available for selection, with multiple responses possible. Responses could be categorized into four areas: Information from the health care system, from the community, from the sports clubs, and from friends/acquaintances. The questionnaire was sent to 394 persons and 133 (34%) completed it (mean age 56 ± 9 years, 73% women).

**Results:**

One health insurance company sent letters to its insured, which was the most successful strategy (mentioned by 33 participants = approximately 25%). The second best strategy is word of mouth through friends/acquaintances (28 participants). Other good recruitment strategies are the community newspaper (23 participants), and activities of the sports club trainer (19 participants). 437 general practitioners working within a 10 km radius of a Jackpot.fit course were informed in writing about Jackpot.fit and were asked to inform their inactive patients participating in Jackpot.fit. However, of the 133 participants, only 3 indicated that their primary care physician informed them about the program.

**Conclusion:**

Health insurance companies can successfully recruit physically inactive adults with a letter to a standardized sports club program near their home. Community information channels and word of mouth have also been successful. Although the network of general practitioners working in the region is dense, written information about the program was not a successful recruitment strategy. Therefore, different recruitment approaches by different actors seem to work best.

